# Evaluation of difficult laryngoscopy with ultrasonography in pediatric patients: Prospective study

**DOI:** 10.1097/MD.0000000000047776

**Published:** 2026-02-28

**Authors:** Osman Kaya, Sema Şanal, Meryem Onay

**Affiliations:** a Department of Anesthesiology and Intensive Care, Southend University Hospital, Essex, United Kingdom; b Department of Anesthesiology and Reanimation, SBU Haydarpaşa Numune Training and Research Hospital, İstanbul, Turkey; c Department of Anesthesiology and Reanimation, Eskişehir Osmangazi University Faculty of Medicine, Eskişehir, Turkey.

**Keywords:** airway ultrasonography, difficult airway, difficult laryngoscopy, pediatric airway, ultrasonography

## Abstract

Effective airway management is a critical aspect of anesthesia, with difficult laryngoscopy (DL) posing significant challenges, particularly in pediatric patients, due to anatomical and physiological differences. This study aimed to evaluate the utility of ultrasonographic measurements of the upper airway as predictors of DL in children. Pediatric patients aged 2 to 8 years who underwent endotracheal intubation under general anesthesia were included. According to the Cormack–Lehane classification (CL), grades 3 and 4 are defined as difficult laryngoscopies. Preoperative ultrasonographic measurements of the hyoid bone-to-skin distance (DSHB), epiglottis-to-skin distance (DSE), and vocal cord anterior commissure-to-skin distance (DSAC) were obtained. The DSAC/DSE ratio was calculated and its potential for predicting DL was assessed. A total of 121 pediatric patients were included in the final analysis, and 6 patients had DL. This study found no correlation between ultrasonographic measurements and the difficulty of laryngoscopy, as classified by the CL score. However, the Mallampati score was a predictor of DL and showed a positive correlation with higher difficulty grades. In this study, the effectiveness of DSHB, DSAC, DSE measurements, and the DSAC/DSE ratio in predicting DL in pediatric patients was evaluated; however, no significant correlation was found. Further research is needed to validate these findings and improve predictive models for difficult pediatric airway management.

## 1. Introduction

Effective airway management is the cornerstone of anesthesia practice. Inadequate airway management remains a significant contributor to anesthesia-related mortality and morbidity.^[1–3]^ Advancements in airway management, including techniques such as laryngeal masks, fiberoptic intubation, videolaryngoscopy, and pre-oxygenation, have significantly reduced mortality and morbidity rates in anesthesia practice.^[4]^ Despite these advancements, the identification and management of difficult intubation continue to pose significant challenges in anesthesia practice. Although traditional physical findings and measurement techniques are recommended for predicting difficult intubation, their reliabilities remains inconsistent.^[5]^ Under these circumstances, only 50% of difficult airway cases can be reliably predicted during the preoperative assessment.^[6]^

In pediatric patients, anatomical and physiological differences add complexity to airway management. This population requires the use of more detailed and tailored approaches to identify and manage difficult intubation.^[7]^ Recent years have seen a growing body of literature indicating that specific upper airway ultrasound measurements may improve the prediction of difficult airways.^[8]^ Some studies have focused on the ratios or differences between these measurements; however, these have predominantly been conducted in adult patients. In these studies, parameters such as skin to hyoid (DSH) + epiglottis (DSE), DSE- skin to glottis (DSG), pre-epiglottic space (PE)/ the epiglottis to the vocal cords (E-VC) ratio, and PES/EVC ratio were investigated. One study, it was emphasized that the use of DSE and DSE-DSG together with classical parameters could provide better information for defining difficult laryngoscopy (DL).^[9–11]^ In studies involving pediatric patients, different age groups have yielded varying outcomes, and there is no clarity on whether these parameters can be used for children.^[12]^ This study sought to utilize the growing accessibility of ultrasonography and its incorporation into routine practice to explore upper airway measurements and their ratios in the pediatric population. By fostering novel approaches and methods, it aims to enhance anesthesia safety and increase success rates in managing difficult airway cases through the early identification and management of DL in pediatric patients.

This study aimed to evaluate the accuracy of preoperative ultrasound assessment of the hyoid bone-to-skin distance (DSHB), epiglottis-to-skin distance (DSE), vocal cord anterior commissure-to-skin distance (DSAC) measurements, and DSAC/DSE ratio in predicting DL in pediatric patients undergoing elective surgery under general anesthesia.

## 2. Method

This prospective study was conducted in the operating theaters of Eskisehir Osmangazi University Faculty of Medicine Hospital between May 2023 and December 2023 and was approved by the Eskisehir Osmangazi University Non-Interventional Clinical Research Ethics Committee (Ethics Committee Approval Number: 2022/12). The protocol for this study was recorded in an international registry, ClinicalTrials.gov, under the registration number NCT05833347.

Before the commencement of the study, written informed consent was obtained from the parents of all participants following comprehensive briefing. The study enrolled 121 pediatric patients aged 2 to 8 years scheduled for elective surgery under general anesthesia with endotracheal intubation and categorized as American Society of Anesthesiologist physical status I to II. Patients were excluded if they had syndromic conditions, congenital maxillofacial anomalies, tracheal or laryngeal abnormalities, pulmonary disorders such as bronchial asthma or airway hyperreactivity, an allergy to ultrasound gel, body weight outside the 10th to 90th percentiles, a history of difficult intubation, or required emergency surgery.

Preoperative evaluation of the patients was conducted at the anesthesia clinic. Routine laboratory tests (complete blood count, biochemistry, and coagulation parameters) were reviewed and physical examinations, including airway assessment and Mallampati classification, were performed. Demographic data such as age, weight, height, sex, body mass index (BMI), and American Society of Anesthesiologists physical status classification scores were recorded.

In this study, the patients were divided into 2 groups according to the modified Cormack–Lehane classification (CL). Grade 1 (complete visibility of the glottis), 2a (partial visibility of the vocal cords), and 2b (visibility limited to the arytenoids or the posterior origins of the vocal cords) were defined as easy laryngoscopy, while grade 3 (only the epiglottis visible) and 4 (no glottic structures visible) were defined as DL.

**Figure 1.**
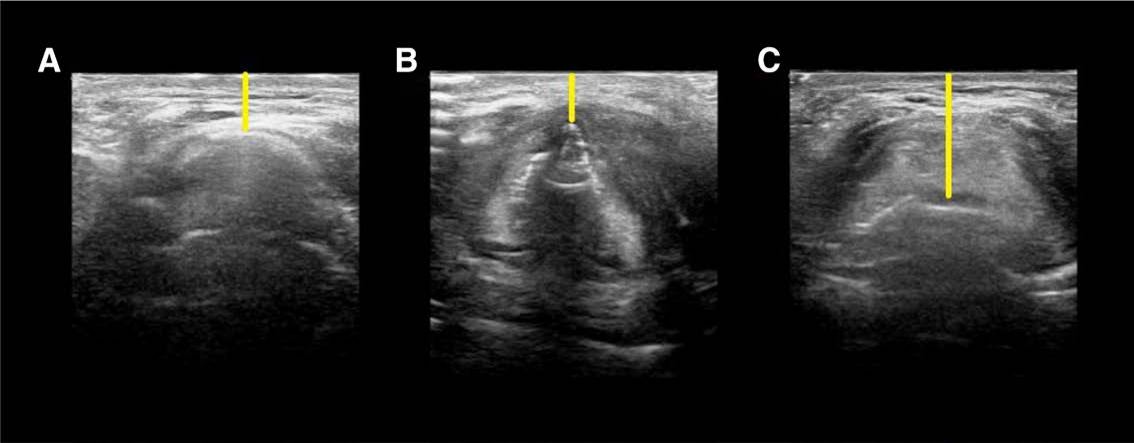
Ultrasound images of the measurements. Ultrasound distances: (A) DSHB, distance between the hyoid bone and skin; (B) DSAC, distance between the vocal cord anterior commissure and skin; (C) DSE, distance between the epiglottis and skin.

In pediatric patients, premedication was administered with midazolam at a dose of 0.3 mg/kg orally or 0.1 mg/kg intravenously. Ultrasound measurements were performed in the preoperative waiting area for patients in whom full communication could be established and in the presence of their parents for those with whom communication was not possible. Ultrasonographic imaging was conducted with the patient in a supine position and the head in neutral alignment using a linear transducer with a frequency range of 6 to 13 Hz (Philips Affiniti 50, Philips Medical Systems, Seattle). A transducer was used to scan the caudal submental region in the transverse plane. The DSHB, DSE, and DSAC were measured (Fig. 1). Based on these values, DSAC/DSE ratios were calculated and recorded. All measurements were performed by a single operator (O.K.) with expertise in ultrasonography.

Following ultrasonographic evaluation, anesthesia was induced in patients with existing intravenous access. Standard monitoring, including electrocardiography, peripheral oxygen saturation, and noninvasive blood pressure, was performed while the patients were in the supine position. In patients without intravenous access, induction was initiated by using a mask with sevoflurane, after which an appropriately sized intravenous line was established. For anesthesia induction, 2 mg/kg propofol, 0.6 mg/kg rocuronium, and 1 mcg/kg remifentanil were administered. Intubation was performed using a Macintosh metal blade of an appropriate size and an endotracheal tube by an anesthesiologist with a minimum of 2 years of pediatric anesthesia experience. The anesthesiologist was blinded to the ultrasonographic findings and independent of the operator performing the ultrasound. The glottic structures observed during laryngoscopy were classified using the modified CL system. In cases of difficult intubation, the anesthesiologist adhered to the standard algorithm.

The groups were labeled as Group E for easy laryngoscopy and Group D for DL. Beyond the comparison between the easy and DL groups, statistical analyses were conducted for each CL grade.

### 2.1. Sample size and statistical analysis

Owing to the lack of prior studies investigating these specific ultrasonographic parameters in the pediatric population and the potential inaccuracy of extrapolating data from adult studies, a direct reference for effect size calculation was not available. Therefore, the sample size was calculated based on a standardized effect size of 0.25 (accepted as a small-to-medium effect according to Cohen conventions) to ensure sufficient statistical power to detect significant differences. Using G*Power software, with a type I error (α) of 0.05 and statistical power (1–β) of 80%, a total of 120 patients were included.

Continuous variables were reported as mean ± standard deviation or median (Q1–Q3), and categorical variables were presented as percentages (%). The Shapiro–Wilk test was used to evaluate the normality of the data distribution. For comparisons of non-normally distributed data, the Mann–Whitney *U* test was applied when there were 2 groups, whereas the Kruskal–Wallis *H* test was used for 3 or more groups. Analysis of contingency tables was performed using Pearson χ^2^ test and Pearson exact χ^2^ test. The Spearman correlation coefficient was calculated to assess the direction and strength of the relationship between the ordinal variables of the Mallampati score and Cormack–Lehane score. Statistical analyses were performed using the IBM SPSS Statistics 21.0 (IBM Corp. Released 2012. IBM SPSS Statistics for Windows, Version 21.0, IBM Corp., Armonk). Statistical significance was set at *P* < .05.

## 3. Results

The data of 121 patients who underwent elective surgery under general anesthesia were prospectively analyzed. Based on laryngoscopic findings, 115 patients were categorized as having easy laryngoscopy, while 6 patients were classified as having DL.

The mean age of all patients was 4.65 ± 1.87 years, the mean weight was 19.25 ± 5.79 kg, the mean height was 108.77 ± 13.74 cm, and the mean BMI was 15.91 ± 1.51 kg/m^2^. The American Society of Anesthesiologists classification was I/II in 99 (82%) and 22 (18%) patients. The distribution of the modified Mallampati score (MMS) was 73 (60%) for grade 1, 47 (39%) for grade 2, and 1 (1%) for grade 3.

An analysis was also conducted based on the CL classification subgroups. Patients were categorized into 4 groups: CL I, CL IIa, CL IIb, and CL III. No case of CL IV was identified in this study. Demographic characteristics did not differ significantly among the CL subgroups (*P* > .05), with the exception of the MMS, which demonstrated a significant correlation (*P* < .05). A positive correlation was found between higher MMS grades and CL classifications (*R* = 0.30, *P* < .001). The demographic data across the CL subgroups are presented in Table 1.

**Table 1:** The relationship between patients’ demographic data and subgroups.

Parameter	I (n = 38)	IIa (n = 44)	IIb (n = 33)	III (n = 6)	*P*-value
Age (yr)	4.24 ± 1.79	4.82 ± 1.90	4.91 ± 1.91	4.67 ± 1.97	–
Weight (kg)	17.89 ± 5.97	19.96 ± 6.08	19.71 ± 4.92	20.17 ± 6.85	–
Height (cm)	105.63 ± 14.29	109.98 ± 13.35	110.76 ± 13.48	108.83 ± 14.39	–
Gender					
Male	29 (%77)	29 (%66)	28 (%85)	4 (%66)	–
Female	9 (%23)	15 (%34)	5 (%15)	2 (%34)	–
ASA					
I	32 (%84)	36 (%82)	26 (%79)	5 (%83)	–
II	6 (%16)	8 (%18)	7 (%21)	1 (%17)	–
MMS					
1	29 (%77)	28 (%64)	14 (%42)	2 (%33)	.049*
2	9 (%23)	16 (%35)	18 (%55)	4 (%67)	
3	0	0	1 (%3)	0	

ASA = American Society of Anesthesiologists, BMI = body mass index, MMS = modified Mallampati score.

* *P* < .05 was considered statistically significant.

A comparison between groups E and D revealed no significant differences in the demographic characteristics or airway parameters (*P* > .05). Although slight variations were noted in the ultrasound measurements between the 2 groups, none were statistically significant. The ultrasound measurement results for both the groups are presented in Table 2.

**Table 2:** Comparison of ultrasound measurements between groups E and D.

Parameter	Group E (n = 115)	Group D (n = 6)	*P*-value
DSHB (cm)	0.49 ± 0.13	0.50 ± 0.14	.73
DSAC (cm)	0.60 ± 0.10	0.66 ± 0.09	.16
DSE (cm)	1.49 ± 0.18	1.61 ± 0.23	.14
DSAC/DSE	0.41 ± 0.06	0.41 ± 0.03	.90

DSAC = vocal cord anterior commissure-to-skin distance, DSE = epiglottis-to-skin distance, DSHB = hyoid bone-to-skin distance.

Ultrasound measurements and the DSAC/DSE ratio, proposed as potential predictors of DL, showed no statistically significant differences between the CL subgroups (*P* > .05). Ultrasound measurement results for the subgroups are shown in Table 3.

**Table 3:** Ultrasound measurements across CL subgroups.

Parameter	CL I (n = 38)	CL IIa (n = 44)	CL IIb (n = 33)	CL III (n = 6)	*P*-value
DSHB (cm)	0.48 ± 0.14	0.52 ± 0.13	0.45 ± 0.11	0.50 ± 0.14	.18
DSAC (cm)	0.60 ± 0.11	0.61 ± 0.10	0.60 ± 0.08	0.66 ± 0.09	.52
DSE (cm)	1.46 ± 0.17	1.51 ± 0.18	1.49 ± 0.17	1.61 ± 0.23	.22
DSAC/DSE	0.41 ± 0.05	0.41 ± 0.07	0.40 ± 0.06	0.41 ± 0.03	.92

DSAC = vocal cord anterior commissure-to-skin distance, DSE = epiglottis-to-skin distance, DSHB = hyoid bone-to-skin distance.

**Figure 2.**
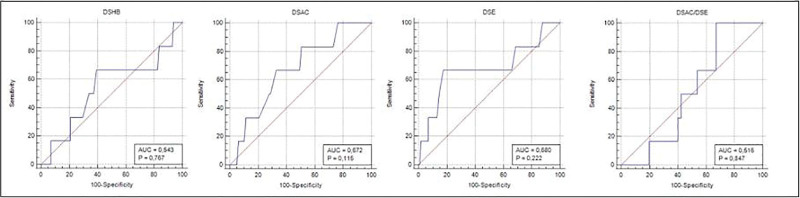
ROC analysis of the ultrasound measurements and Cormack–Lehane. In the charts obtained with ROC analysis of the 3 ultrasound measurements, Cormack–Lehane. DSHB = distance from the hyoid bone to the skin surface, DSAC = distance from the skin to the anterior commissure of the vocal cords, DSE = distance from the skin to the epiglottis midway, DSAC/DSE = ratio of the distance from the skin to the anterior commissure of the vocal cords to the distance from the skin to the epiglottis, ROC = receiver operating characteristic.

Receiver operating characteristic analysis was performed to evaluate the clinical ability of the measured ultrasonographic distances to discriminate between easy and difficult laryngoscopies. DL was defined as CL grade ≥ III, whereas grades I to II were considered easy laryngoscopy. The receiver operating characteristic analysis charts revealed similar results in identifying the cutoff points for predicting DL based on the measured distances. The areas under the curve for the various ultrasound measurements were as follows: DSHB = 0.543, DSAC = 0.672, DSE = 0.680, and DSAC/DSE = 0.516. No significant differences were observed in pairwise comparisons of these measurements (*P* > .05) (Fig. 2).

## 4. Discussion

This prospective study aimed to assess the utility of neck ultrasonographic measurements in predicting DL in pediatric patients aged 2 to 8 years and demonstrated that the Mallampati score was successful in predicting the CL score. Furthermore, preoperative ultrasonographic evaluations included measurements of the DSHB, DSE, DSAC, and the DSAC/DSE ratio. However, no statistically significant correlation was observed between these measurements and the easy and DL groups categorized according to the CL classification.

The variability in the incidence of difficult intubation, reported to range from 5 to 22%, can be attributed to the use of different assessment tests, variations in practitioner experience, and differences in population characteristics.^[13–15]^ In pediatric patients, despite the limited availability of large-scale studies, the incidence of difficult direct laryngoscopy (DL) has been reported as 1.35% with an experienced team. It has been emphasized that the prediction of DL is reliable if the Mallampati score is obtained.^[16]^ While the incidence of DL was 4.8% in the study by Zheng et al, an unexpected difficult airway was observed in 4.95% of the cases in our study.^[12]^ However, the primary aim of this study was not to determine the incidence of DL and the sample size was too small to calculate it.

The Mallampati score, a commonly used test for predicting DL, was first published by Mallampati et al in 1985 and later modified by Samsoon and Young in 1987.^[17,18]^ The MMS has become one of the most widely used tools for predicting difficult airways owing to its simplicity and clear assessment criteria. Nevertheless, the accuracy of airway evaluation using MMS can be significantly influenced by its dependence on patient cooperation and correct positioning.^[19]^ Therefore, MMS has been reported to be a reliable predictor in numerous studies, yet some research has indicated that its predictive value may be limited.^[20]^ A recent meta-analysis of 177,088 patients revealed that MMS is inadequate as a standalone tool for predicting DL or tracheal intubation. Nevertheless, it can contribute to a multifactorial model for predicting difficult tracheal intubation.^[21]^ Although our study included pediatric patients, considering that the position may be variable, a significant correlation was observed between higher Mallampati scores and higher CL scores.

In recent years, the growing perioperative use of ultrasonography has led to numerous studies aimed at predicting DL using airway ultrasound. These studies have shown that specific ultrasound parameters, including tongue base thickness,^[22]^ hyomental distance in extension (DEHM),^[23]^ DSE,^[24]^ tongue volume,^[25,26]^ mid-sagittal tongue cross-sectional area, and tongue width,^[23,27]^ are effective in predicting difficult airway in adults. A meta-analysis by Carsetti et al demonstrated that DSE was an effective predictor of DL in an adult population. In their study, which reviewed 10 studies involving 1812 patients, a higher DSE value was observed in patients with a high CL grade.^[28]^ Wu et al’s prospective observational study involved 203 patients aged 20 to 65 years, in which a strong positive correlation was identified between DSE, DSHB, DSAC, and intubation difficulty.^[29]^ Adhikari et al measured soft tissue in the anterior neck at the level of the hyoid bone and thyrohyoid membrane, which are closely related to the vallecula that needs to be raised during laryngoscopy. Ultrasonographic measurements have demonstrated that the thickness of the soft tissue in the anterior neck at the level of the hyoid bone and thyrohyoid membrane can be used to predict DL, with a 2.8 cm ultrasound measurement at the thyrohyoid membrane serving as a strong independent predictor for DL.^[30]^ In our study, no significant differences were observed in the demographic data of patients with a BMI within the normal limits. Furthermore, no significant correlation was found between preoperative ultrasonographic measurements of DSHB, DSE, and DSAC and DL.

While several studies conducted in adult populations have demonstrated favorable predictive performance of ultrasonographic airway parameters such as DSE, DSAC, DSHB, and related ratios, findings in pediatric populations have been inconsistent. Adult airway anatomy is relatively stable, and increased anterior neck soft tissue thickness has been shown to contributes to DL. In contrast, pediatric airway anatomy is characterized by continuous growth, variable tissue compliance, and age-dependent anatomical proportions, which may limit the applicability of adult-derived ultrasound cutoff values. In pediatric studies, some authors have reported that specific parameters, particularly DSE, may predict DL in selected age groups, whereas others have failed to demonstrate reliable discrimination.^[12]^ Our findings are consistent with these latter studies, suggesting that ultrasonographic measurements alone may not provide sufficient predictive accuracy in children aged 2 to 8 years.

The primary goal of these studies was to identify cases of difficult intubation for which traditional methods proved inadequate. Current tests exhibit low sensitivity for detecting difficult intubation.^[31]^ Syndromic conditions, an enlarged and protruding tongue, micrognathia, and a high palate are potential signs of difficult intubation in pediatric patients.^[32]^ They were excluded from the study.

In a prospective observational study by Martínez-García et al involving 50 adult patients, attention was paid to the connection between the epiglottis and DSG (glottis). It was shown that an increase in the difference between DSE and DSE-DSG could significantly predict difficult intubation. This was the only study identified in our literature review that demonstrated the potential of DSE-DSG difference as a predictor of DL. The epiglottis is suspended from the hyoid bone by the hyoepiglottic ligaments. During laryngoscopy, when the epiglottis is lifted with the vallecula, if the expected glottis appears to be anteriorly positioned, the glottis may be located closer to the skin.^[9]^ In a study conducted by Mohammadi et al with 72 adult patients, the correlation between DSE and the ratio of the mid-vocal cord-to-skin distance was evaluated. They found a weak correlation between the ratio and CL grade, with 87% sensitivity and 30% specificity.^[10]^ In a study by Yildiz et al, involving 136 adult patients, the same ratio was examined, and the results showed a weak correlation with the CL classification.^[11]^

Our literature review found no studies investigating a similar ratio in pediatric patients. The pediatric patient group in our study consisted of individuals with varying weights and heights that changed with age. Furthermore, because of differences in the rate of physical development within the same age group, we believe that investigating a single measurement cutoff value, as in adult patients, would be misleading. Therefore, we considered the ratio between DSE and DSAC, rather than the difference, to be more significant. In our study, no statistically significant relationship was found between DSAC/DSE ratio and DL.

Ultrasound studies predicting DL have typically focused on the subcutaneous thickness of the anterior neck in adults.^[8,26,28,33]^ This has led to a focus on studies primarily involving obese patients, and it has been shown that the depth of airway structures, such as DSE and DSAC distance, may be predictive of DL.^[34]^ Although there is a need for an easily applicable test with high positive and negative predictive values in pediatric patients, such studies are rare in the pediatric population in contrast to those in adult patients. In a study by Zheng et al, which involved 358 pediatric patients aged 5 to 12 years, an increased DSE could be used to predict DL in the 5 to 8-year-old group. However, in the 9 to 12-year-old age group, DSE’s predictive value of DSE was not statistically significant.^[12]^ The variability of the relationship between DSE and DL in different age groups and the inclusion of children under 5 years of age, for whom measurements are more difficult, in our study may explain the differences in the results.

Children aged > 2 years were included in the present study. Children under 2 years of age were excluded because of anatomical differences and variations, such as the recommendation to place an elevation under the shoulders during intubation. After the age of 8 years, pediatric airway anatomy begins to resemble adult anatomy, and since there are already existing publications regarding airway management in adults, we included only children older than 2 years in this study to standardize airway anatomy.

Studies have considered DL as a CL 3 to 4. Some studies stating that additional measures are required during management have also classified CL 2b as DL.^[33,34]^ In our study, we considered that CL 2b could result in changes to airway management, such as the use of a stylet or bougie, and therefore analyzed all CL groups separately, apart from the difficult and easy laryngoscopy groups. Similar to the difficult and easy laryngoscopy groups, no statistically significant differences were found in our measurements between the groups. This may be because of the low incidence of DL, leading to a smaller number of patients in these groups.

Several studies have evaluated the difference between DSE and DSG as well as the sum of DSE and DSG, along with other values, for predicting DL. In addition, studies have examined the DSAC/DSE ratio in adult patients. We believe that our study contributes to the literature by evaluating the DSAC/DSE ratio for the first time for predicting DL in pediatric patients.

Our study has several limitations. Direct visualization of the glottis with a laryngoscope is a complex task influenced by numerous subjective and objective factors, such as the operator skill and experience, airway secretions, and anatomical abnormalities. Consequently, the small sample size may have limited the generalizability of our findings. Given the low incidence of DL and the cross-sectional nature of the study, it was not feasible to have an equal number of patients in both the groups. Consequently, the difficult group had 6 patients, while the easy group included 115 patients.

This was a single-center study, which may limit the generalizability of the results. Another factor is that the child must keep their head as still as possible while awake during the measurement process. The linear transducer must be placed over the thyrohyoid membrane to measure the distance between the skin and epiglottis, and the weight of the transducer may cause discomfort in children. This is especially true for children under 5 years of age, as they are generally less cooperative, which increases the likelihood of errors during the measurements.

One of the challenges faced while performing airway ultrasonography in the pediatric population is that the pressure exerted by the transducer during application influences the measured values because of the small and soft tissues. In our study, all the patients were evaluated by a single clinician who had undergone airway ultrasonography. Likewise, to eliminate inter-operator variability, all intubations were performed by an anesthesiologist with a minimum of 2 years of pediatric anesthesia experience. Ultrasound measurements in children, which require time and are immobile, are more challenging to perform than those in adults.

In conclusion, this study, in addition to the DSE, DSAC, and DSHB measurements, investigated the DSAC/DSE ratio, an ultrasound measurement that has not been explored in pediatric patients. Although we did not find a significant correlation with DL, we believe that this research is important as it may guide future studies exploring new ultrasound-based predictive factors for pediatric airway management.

## Acknowledgments

This study has been registered at ClinicalTrials.gov (NCT05833347).

## Author contributions

**Conceptualization:** Osman Kaya, Sema Şanal.

**Data curation:** Osman Kaya, Meryem Onay.

**Methodology:** Sema Şanal, Meryem Onay.

**Supervision:** Sema Şanal, Meryem Onay.

**Writing – original draft:** Osman Kaya.

**Writing – review & editing:** Sema Şanal, Meryem Onay.
